# Growth Hormone Withdrawal in Mid-Puberty: No Impact on Near Adult Height in Adolescents With Transient Idiopathic GHD

**DOI:** 10.1210/clinem/dgaf626

**Published:** 2025-11-15

**Authors:** Joeri Vliegenthart, Jan M Wit, Boudewijn Bakker, Annemieke M Boot, Christiaan de Bruin, Martijn J J Finken, Josine C van der Heyden, Anita C S Hokken-Koelega, Hetty J van der Kamp, Edgar G van Mil, Theo C J Sas, Dina A Schott, Petra van Setten, Saartje Straetemans, Vera van Tellingen, Robbert N H Touwslager, A S Paul van Trotsenburg, Paul G Voorhoeve, Edmond H H M Rings, Erica L T van den Akker, Danielle C M van der Kaay

**Affiliations:** Division of Pediatric Endocrinology, Department of Pediatrics, Erasmus University Medical Centre, Sophia Children's Hospital, 3015 GD Rotterdam, the Netherlands; Division of Pediatric Endocrinology, Department of Pediatrics, Willem-Alexander Children's Hospital, Leiden University Medical Centre, 2333 ZG Leiden, the Netherlands; Division of Pediatric Endocrinology, Department of Pediatrics, Reinier de Graaf Gasthuis, 2625 AD Delft, the Netherlands; Division of Pediatric Endocrinology, Department of Pediatrics, Beatrix Children's Hospital, University Medical Centre Groningen, 9713 GZ Groningen, the Netherlands; Division of Pediatric Endocrinology, Department of Pediatrics, Willem-Alexander Children's Hospital, Leiden University Medical Centre, 2333 ZG Leiden, the Netherlands; Division of Pediatric Endocrinology, Department of Pediatrics, Emma Children's Hospital, Amsterdam University Medical Center, 1105 AZ Amsterdam, the Netherlands; Division of Pediatric Endocrinology, Department of Pediatrics, Franciscus Gasthuis & Vlietland, 3045 PM Rotterdam, the Netherlands; Dutch Growth Research Foundation, 3016 AH Rotterdam, the Netherlands; Department of Pediatrics, Erasmus University Medical Centre, Sophia Children's Hospital, 3015 GD Rotterdam, the Netherlands; Division of Pediatric Endocrinology, Department of Pediatrics, Wilhelmina Children's Hospital, University Medical Center Utrecht, 3584 CX Utrecht, the Netherlands; Division of Pediatric Endocrinology, Department of Pediatrics, Jeroen Bosch Hospital, 5223 GZ ‘s-Hertogenbosch, the Netherlands; Division of Pediatric Endocrinology, Department of Pediatrics, Erasmus University Medical Centre, Sophia Children's Hospital, 3015 GD Rotterdam, the Netherlands; Division of Pediatric Endocrinology, Department of Pediatrics, Zuyderland Hospital, 6419 PC Heerlen, the Netherlands; Division of Pediatric Endocrinology, Department of Pediatrics, Amalia Children's Hospital, Radboud University Medical Centre, 6525 GA Nijmegen, the Netherlands; Division of Pediatric Endocrinology, Department of Pediatrics, Maastricht University Medical Centre, 6229 HX Maastricht, the Netherlands; Division of Pediatric Endocrinology, Department of Pediatrics, Catharina Hospital, 5623 EJ Eindhoven, the Netherlands; Division of Pediatric Endocrinology, Department of Pediatrics, St. Antonius Hospital, 3543 AZ Nieuwegein, the Netherlands; Division of Pediatric Endocrinology, Department of Pediatrics, Emma Children's Hospital, Amsterdam University Medical Center, 1105 AZ Amsterdam, the Netherlands; Division of Pediatric Endocrinology, Department of Pediatrics, Canisius Wilhelmina Hospital, 6532 SZ Nijmegen, the Netherlands; Department of Pediatrics, Erasmus University Medical Centre, Sophia Children's Hospital, 3015 GD Rotterdam, the Netherlands; Division of Pediatric Endocrinology, Department of Pediatrics, Erasmus University Medical Centre, Sophia Children's Hospital, 3015 GD Rotterdam, the Netherlands; Division of Pediatric Endocrinology, Department of Pediatrics, Erasmus University Medical Centre, Sophia Children's Hospital, 3015 GD Rotterdam, the Netherlands

**Keywords:** idiopathic isolated growth hormone deficiency (IIGHD), GH retesting, mid-puberty, rhGH withdrawal, near adult height (NAH)

## Abstract

**Context:**

In children with idiopathic isolated growth hormone deficiency (IIGHD), GH secretion often normalizes by near adult height (NAH). Whether recombinant human GH (rhGH) treatment can be safely discontinued earlier remains unclear.

**Objective:**

This work aimed to investigate if withdrawing rhGH treatment from mid-puberty onward had no negative effect on attained NAH in adolescents who, after retesting, were no longer GH deficient.

**Methods:**

A prospective multicenter patient preference study was conducted at pediatric endocrinology departments in multiple centers (2017-2024) with follow-up until NAH (SEENEZ GH Study). Participants included 127 adolescents (95 male, 75%) with childhood IIGHD (GH peak 1.7-10 µg/L) who tested GH sufficient (GH peak >6.7 µg/L) at mid-puberty. Forty-four continued rhGH (GHcont), 83 discontinued (GHstop). A total of 99% of patients completed the study. Intervention included rhGH treatment continuation vs discontinuation from mid-puberty until NAH. The primary outcome measure was NAH-SDS minus target height (TH)-SDS. The secondary outcome was NAH-SDS, total pubertal growth (TPG), and predicted vs attained height gain.

**Results:**

Mean (SD) NAH-SDS minus TH-SDS was −0.17 (0.60) in the GHcont and −0.18 (0.62) in the GHstop group (*P* = .96). Mean NAH-SDS was −0.91 (0.76) (GHcont) vs −0.78 (0.76) (GHstop) (*P* = .35). Mean (SD) TPG (from start of puberty) in males was 27.5 cm (7.0; GHcont) vs 25.9 cm (6.2; GHstop) (*P* = .25) and in females 20.5 cm (5.7; GHcont) vs 20.9 cm (7.6; GHstop) (*P* = .90). Predicted vs attained height gain based on the prediction model did not differ between groups.

**Conclusions:**

In adolescents with transient IIGHD, rhGH treatment can be stopped at mid-puberty. These findings support reducing rhGH treatment duration, lowering patient burden and health-care costs.

In the Netherlands, each year approximately 100 to 125 children with growth hormone deficiency (GHD) start recombinant human growth hormone (rhGH) treatment after a thorough check of clinical, laboratory, and radiological features. About half of these children are diagnosed with idiopathic isolated GHD (IIGHD). The main aim of rhGH treatment is to reach a normal adult height (AH) defined as height within the normal AH range for the population, as well as within the range of the sex-corrected mid-parental height (target height, TH ± 1.6 SDS). rhGH treatment is usually continued until near AH (NAH) is reached (defined as growth velocity <2 cm/year). A growth rate of <15 mm over a 6-month period suggests that growth is likely to stop within 2 years (1-3). Thereafter, patients with GHD are retested to establish if rhGH treatment needs to be continued in adulthood. It is recommended that adult patients with GHD continue rhGH treatment, as untreated GHD is associated with increased risks of metabolic complications such as diabetes, osteoporosis, and dyslipidemia, while treatment improves body composition, muscular strength, bone health, and quality of life (4, 5).

When patients with IIGHD are retested at (N)AH, around 60% to 70% of them show a normal GH peak (6-9), in contrast to almost none of the patients with multiple pituitary hormone deficiency or isolated GHD due to organic or genetic causes, who therefore generally do not require retesting. It is assumed that in IIGHD cases the initial test result may have been false positive, or that GHD may be transient (10). Both explanations may be associated with the physiological increase of GH secretion due to endogenous sex steroids in puberty (11, 12). Moderate agreement between GH stimulation tests (GHSTs) suggests that test variability may lead to misclassification, indicating that some children diagnosed with IIGHD may not have true GHD (13-15).

The general belief is that the main effect of rhGH on AH is obtained before puberty, particularly in the first 2 to 3 years after starting rhGH (16-20). The efficacy of rhGH treatment during the latter half of puberty in patients diagnosed with IIGHD in childhood remains an unsolved question (21). It appears plausible that, if normal GH secretion is observed in mid-puberty, rhGH treatment may not affect AH. In 2 partly controlled studies aimed at answering this question, it was suggested that discontinuation of rhGH treatment in mid-puberty would not lead to a significant reduction in AH (9, 21). We hypothesized that withdrawing rhGH treatment from mid-puberty onward would not have a negative effect on attained NAH in adolescents who are no longer GH-deficient on retesting.

## Materials and Methods

### Study Design

We performed a multicenter, prospective patient preference design study. A randomized controlled trial (RCT) was not deemed feasible after a pilot study in 4 centers in the Netherlands found that approximately 50% of patients would not be willing to participate in an RCT (unpublished data).

All adolescents diagnosed with IIGHD in childhood underwent retesting at mid-puberty at least 1 month after rhGH treatment discontinuation, according to the national treatment protocol (22). Subsequently, those with a normal test were offered the option to either continue rhGH treatment until NAH would be attained (traditional approach) or to discontinue rhGH treatment from mid-puberty onward.

### Patients

The study population consisted of adolescents in mid-puberty who started rhGH treatment after the diagnosis of partial IIGHD was made and were treated for at least 3 years. Partial IIGHD was defined as the highest GH peak in 2 GHSTs between 1.7 and 10 µg/L (5-30 mU/L). GHD was classified according to the Dutch national protocol developed by the Growth Hormone Advisory Group, which is based on GH peak during stimulation tests and serum insulin-like growth factor-I (IGF-I), and includes 7 categories, with categories 1 to 4 considered candidates for rhGH treatment (23, 24). At diagnosis, various GHSTs were used. At mid-puberty, most patients underwent the same tests as at diagnosis; in 11 cases, 1 of the 2 tests was repeated combined with another test, while in 3 cases the growth hormone–releasing hormone (GHRH) + arginine test was used instead of the arginine + clonidine combination. For the first GHST in mid-puberty, the following tests were used: clonidine test (n = 70), arginine test (n = 53), GHRH + arginine test (n = 3), and L-DOPA-propranolol test (n = 1). A second GHST was performed in 78 of 127 patients: clonidine test (n = 32), arginine test (n = 37), L-DOPA-propranolol test (n = 3), glucagon test (n = 3), GHRH + arginine test (n = 3). Five second GHSTs were performed due to an insufficient GH rise during the first test; the remaining second tests were conducted the same day as the first test in accordance with standard protocol.

Mid-puberty in boys was defined as Tanner stage G3 or G4, testicular volume (TV) greater than 12 mL, and a bone age (BA) between 13 and 16 years. In girls, mid-puberty was defined as Tanner stage B3 or B4, and a BA between 11 and 14 years.

Individuals were excluded if they had any medical or mental disorder that could potentially influence growth, or if they were using other medication than rhGH that could potentially influence growth.

Adolescents who exhibited a normal GH peak (7 µg/L = 20 mU/L) on retesting in mid-puberty were eligible for inclusion. Growth and pubertal progression were monitored every 3 to 4 months in the group continuing rhGH treatment (GHcont) and every 6 months in the group who discontinued rhGH treatment (GHstop). If (parents of) children decided to continue rhGH treatment, the treatment was discontinued when NAH was reached.

Two patients had celiac disease that was well controlled with a strict gluten-free diet, ascertained by low antitissue transglutaminase antibodies levels, and therefore assessed as not affecting growth. Another patient underwent genetic testing for a minor abnormality on a hand radiograph. Two genetic mutations related to the abnormality were found (*COL4A1* and *GDF5*). The available literature shows no relation with IIGHD, nor an effect on growth relevant to this patient (25, 26). In consultation with the advisory group members of this study, it was decided not to exclude this patient.

In 3 adopted patients, parental information was lacking and TH could not be calculated. All of these patients were adopted from China. TH in these patients was imputed using the new Chinese growth charts of Zong and Li (27). The median height of 18-year-old boys (172.7 cm) and girls (160.6 cm) was used as TH. TH-SDS was calculated using the Dutch formula and SD values (for 21-year-old males: 7.1 cm and for 21-year-old females: 6.3 cm) (28). Excluding the imputed THs from these 3 patients from the analysis did not significantly affect the primary outcome.

The study was approved by the medical research ethics committee of Amsterdam University Medical Center (protocol No. NL57916.029.16). The study was conducted in accordance with the Declaration of Helsinki (2013) and the Medical Research Involving Human Subjects Act (WMO). Written informed consent was obtained both from patients and their parents.

### Measurements

Height was measured using a stadiometer and reported in centimeters. TV was measured using an orchidometer. Pubertal stage was evaluated using Tanner classification (1). TH was calculated using Dutch references (28). The current TH formula corrects for assortative mating and parent-offspring correlations (29). In the Netherlands, GH levels have been reported in mU/L and µg/L. To use a single unit in the analyses, the values were converted to µg/L. Over the years, different conversion factors have been used by various laboratories. In collaboration with the laboratories and the Dutch Growth Research Foundation, these conversion factors have been mapped and used. IGF-I results have been converted to the SI unit (Système International d’Unités) from ng/mL to nmol/L by multiplying the result by 0.1307 (30, 31).

BA was determined using the Greulich and Pyle method (n = 69) and the BoneXpert method (n = 58) (32-34). When Greulich and Pyle and BoneXpert were both available (n = 7), the BoneXpert method was used.

At the time of diagnosis, all patients received a magnetic resonance imaging scan of the hypothalamic-pituitary region. An ectopic neurohypophysis without further hormonal deficiencies was not considered as the cause of IIGHD. Two patients were diagnosed with mild hypoplasia of the adenohypophysis based on magnetic resonance imaging findings. These 3 patients were included in the study.

### Study End Points

The primary outcome was the comparison of NAH minus conditional TH between the 2 inclusion groups, both expressed as SDS. Secondary outcomes were NAH SDS and total pubertal growth (TPG; height gain in centimeters from Tanner G2 or B2 until NAH).

### Statistical Analysis

Data were analyzed using IBM SPSS Statistics (version 29.0.1.0). The Kolmogorov-Smirnov test was used to assess data distribution. Group comparisons were conducted using the *t* test for normally distributed variables and Mann-Whitney *U* test for nonnormally distributed variables. A 2-tailed *P* value of <.05 was considered statistically significant. SD scores for height, weight, TH, and body mass index (BMI) were calculated using Growth Analyzer RCT, based on data from the Dutch National Growth Studies (35).

We evaluated NAH SDS both at the age when it was measured and at age 21 years to ensure accurate AH SDS calculation (the end point of the Dutch growth charts) (35). This dual reporting helps to avoid significant overestimation of AH SDS caused by early maturation.

It was expected that groups would differ in baseline characteristics (eg, those who chose to discontinue rhGH treatment might be older and taller). Because the number of included patients in the discontinuation group was expected to be too low and the between-group differences too large to show statistically significant “noninferiority” of discontinuing rhGH at mid-puberty, a retrospective analysis was performed. This analysis involved growth, pubertal stages, and BA of a historic control group with IIGHD and normal GH secretion at reevaluation after stopping rhGH treatment at NAH (n = 151). Based on these data, a model was constructed for expected height gain during rhGH treatment from mid-puberty until NAH (36). For both prospectively followed groups in the present study, the expected height gain at inclusion was calculated based on these models. At the end of the observation period, the effectively attained height gain in both groups was compared with the predicted one. We hypothesized that the difference in attained minus predicted height gain in both groups would not be significantly different from zero, and that the 95% CI would exclude a difference greater than 0.5 SD (which would indicate a disadvantage for the group who discontinued rhGH treatment at mid-puberty).

## Results

**Figure 1.**
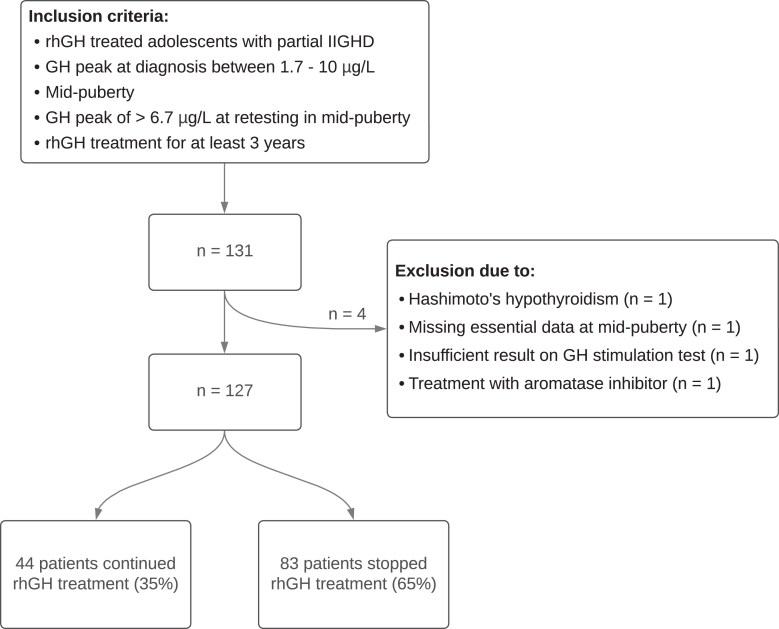
Flowchart illustrating the patient enrollment process, specifying inclusion and exclusion criteria.

Originally, a total of 131 patients were included between 2017 and 2024. Four patients were excluded during the course of the study based on the criteria detailed in Fig. 1, as they were incorrectly included. This resulted in a final cohort of 127 patients for analysis, of whom 44 patients continued rhGH treatment (35%) and 83 patients stopped rhGH treatment (65%). Table 1 presents patient characteristics at study entry. Table 2 gives descriptive statistics of the included patients at the start of rhGH treatment. Boys and girls did not differ in these characteristics.

**Table 1.** Characteristics at mid-puberty

		rhGH continuation group			rhGH stop group			*P*
Sex	n (%)	M	36 (82%)	N = 44	M	59 (71%)	N = 83	.17
		F	8 (18%)		F	24 (29%)		
Age, y	Mean (SD)	M	14.16 (1.03)	N = 44	M	14.18 (1.08)	N = 83	.69
		F	13.07 (0.78)		F	13.05 (0.98)		
TH-SDS	Mean (SD)	−0.74 (0.55)		N = 44	−0.61 (0.54)		N = 83	.15
Height SDS	Mean (SD)	−0.76 (0.69)		N = 44	−0.48 (0.84)		N = 83	.06
BMI-SDS	Mean (SD)	0.07 (1.01)		N = 44	0.03 (1.02)		N = 81	.84
Height SDS minus TH-SDS	Mean (SD)	−0.02 (0.69)		N = 44	0.13 (0.78)		N = 83	.24
Bone age, y	Median (IQR)	M	13.6 (13.3-14.0)	N = 44	M	13.5 (13.0-14.0)	N = 83	.18
		F	12.1 (11.8-12.8)		F	12.0 (11.4-13.0)		
Tanner stage, G/B	n (%)	2	1 (2.4%)	N = 42	2	2 (2.5%)	N = 81	.65
		3	16 (38.1%)		3	32 (39.5%)		
		4	20 (47.6%)		4	41 (50.6%)		
		5	5 (11.9%)		5	6 (7.4%)		
Testicular volume, mL	Median (IQR)	15 (13-18)		N = 36	15 (12-15)		N = 59	.04
GH peak 1st test, µg/L	Median (IQR)	11.2 (7.7-19.0)		N = 44	12.7 (8.6-17.0)		N = 83	.62
GH peak 2nd test, µg/L	Median (IQR)	13.0 (10.7-17.7)		N = 29	15.1 (9.0-21.0)		N = 49	.70
IGF-I, nmol/L	Mean (SD)	45.8 (13.9)		N = 44	48.3 (11.1)		N = 79	.32
Testosterone (M), nmol/L	Median (IQR)	13.1 (11.8-17.4)		N = 8	13.7 (11.0-15.0)		N = 24	.67
Estradiol (F), pmol/L	Mean (SD)	180.5 (57.2)		N = 6	157.9 (87.4)		N = 15	.23

Abbreviations: BMI, body mass index; F, female; GH, growth hormone; IGF-I, insulin-like growth factor-I; IQR, interquartile range; M, male; rhGH, recombinant human growth hormone; SDS, SD score; TH, target height.

**Table 2.** Characteristics at the start of recombinant human growth hormone treatment

		rhGH continuation group			rhGH stop group		
Age, y	Mean (SD)	6.09 (2.25)		N = 44	5.67 (1.89)		N = 83
Height SDS	Mean (SD)	−2.68 (0.57)		N = 44	−2.65 (0.60)		N = 83
Height SDS – TH-SDS	Mean (SD)	−1.95 (0.60)		N = 44	−2.04 (0.53)		N = 83
BMI SDS	Mean (SD)	−0.07 (1.08)		N = 44	−0.12 (1.08)		N = 83
Bone age, y	Median (IQR)	4.13 (3.00-5.78)		N = 40	3.50 (2.67-5.00)		N = 81
Tanner stage (G/B)	n (%)	1	37 (100%)	N = 37	1	72 (100%)	N = 72
GH peak 1st test, µg/L	Mean (SD)	5.6 (2.0)		N = 44	5.7 (1.8)		N = 83
GH peak 2nd test, mU/L	Mean (SD)	5.4 (2.2)		N = 44	5.2 (2.2)		N = 83
IGF-I, nmol/L	Median (IQR)	6.27 (4.44-8.70)		N = 33	5.00 (3.59-7.03)		N = 68

Reference ranges for IGF-I vary between laboratories and are age and sex specific. The 3 centers that contributed the largest number of patients (n = 68) used the following reference intervals: boys aged 5 to 8 years (5-6 years: 6.7-34.2 nmol/L; 6-7 years: 11.5-63 nmol/L; 7-8 years: 6.4-38.2 nmol/L) and girls aged 5-8 years (5-6 years: 4.8-30.4 nmol/L; 6-7 years: 5.9-38.4 nmol/L; 7-8 years: 11.5-63 nmol/L).

Abbreviations: BMI, body mass index; GH, growth hormone; IGF-I, insulin-like growth factor-I; IQR, interquartile range; rhGH, recombinant human growth hormone; SDS, SD score; TH, target height.

Patient characteristics at the start of rhGH treatment are shown in Table 2; variables were comparable between the 2 groups.

**Figure 2.**
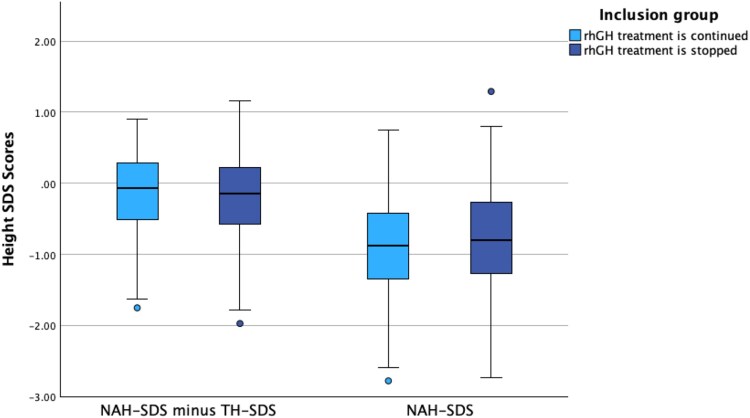
Box plots comparing the group that continued recombinant human growth hormone (rhGH) treatment with the group that discontinued treatment. The left plots show the primary outcome, defined as near adult height–SD score (NAH-SDS) minus target height (TH)-SDS. The right plots show NAH-SDS without correction for TH.

The primary outcome is shown in Fig. 2, which shows 44 participants in the GHcont group and 82 participants in the GHstop group (1 missing case due to being lost to follow-up). The mean NAH-SDS minus TH-SDS in the GHcont group was −.171 (0.60), compared to −0.177 (0.62) in the GHstop group (*P* = .96). The mean difference is 0.006 SDS (0.04 cm in both boys and girls). The mean 21 years-age–adjusted NAH-SDS minus TH-SDS was −0.525 (0.58) in the GHcont group, compared to −0.624 (0.65) in the GHstop group (*P* = .40). The mean difference is 0.099 SDS (0.70 cm in boys and 0.62 cm in girls).

The mean NAH-SDS in the GHcont group was −0.91 (0.76), compared to −0.78 (0.76) in the GHstop group (*P* = .35), with a mean difference of −0.13 SDS. The 21 years-age–adjusted NAH-SDS showed a mean (SD) in the GHcont group of −1.26 (0.74), compared to −1.22 (0.80) in the GHstop group (*P* = .78). The mean difference is −0.04 SDS.

**Figure 3.**
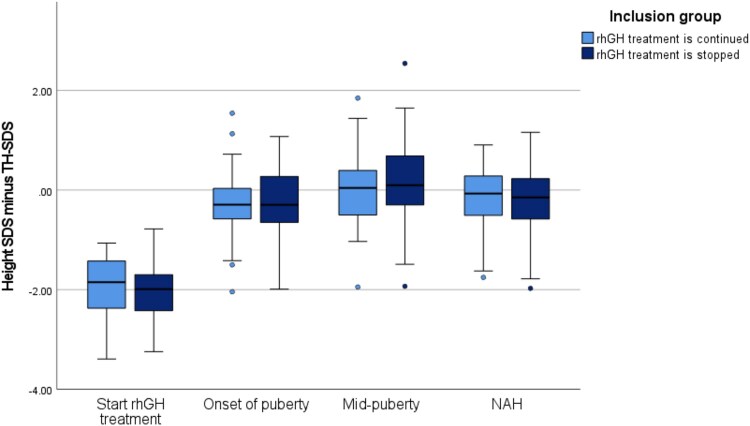
Height SD score (SDS) minus target height (TH)-SDS at start of recombinant human growth hormone (rhGH) treatment, onset of puberty, mid-puberty, and near adult height (NAH). Δ Height SDS was 1.78 (0.66) vs 1.85 (0.60) from start of rhGH treatment to NAH (*P* = .50), 1.92 (0.71) vs 2.17 (0.71) from start to mid-puberty (*P* = .06), and −0.15 (0.50) vs −0.32 (0.49) from mid-puberty to NAH (*P* = .07).

TPG (cm height gain between onset of Tanner G2 or B2 and NAH) was available for 118 patients (93%). The mean (SD) TPG in the GHcont group was 26.10 (7.32) cm, compared to 24.63 (6.89) cm in the GHstop group (*P* = .28). Among boys, the mean (SD) TPG was 27.48 (6.98) cm in the GHcont group and 25.86 (6.22) cm in the GHstop group, with no statistically significant difference (*P* = .25). Among girls, the mean (SD) TPG was 20.51 (5.69) cm in the GHcont group and 20.88 (7.56) cm in the GHstop group (*P* = .90), also showing no significant difference. Growth from mid-puberty until NAH is shown as Δ height SDS in Fig. 3. Δ Height SDS was 1.78 (.66) vs 1.85 (.60) from the start of rhGH treatment to NAH (*P* = .50), 1.92 (0.71) vs 2.17 (0.71) from the start of rhGH treatment to mid-puberty (*P* = .06) and −0.15 (0.50) vs −0.32 (0.49) from mid-puberty to NAH (*P* = .07).

The average duration of rhGH treatment was 10.3 (2.2) years in the GHcont group (10.3 [2.3] years for boys and 10.2 [1.2] years for girls) and 8.2 (1.9) years in the GHstop group (8.4 [1.9] years for boys and 7.8 [1.9] years for girls; *P* < .01).

For boys, the age at last rhGH injection was 16.6 (1.0) years in the GHcont group and 14.2 (1.1) years in the GHstop group (*P* < .001). For girls, the age at last rhGH injection was 15.3 (0.7) years in the GHcont group and 13.1 (1.0) years in the GHstop group (*P* < .001). In the GHcont group, the median age of NAH measurement was 17.8 years (15.6-20.7) for boys and 16.0 years (14.9-21.4) for girls. In the GHstop group, the median age was 17.1 years (14.3-22.29) for boys and 16.0 years (13.9-22.3) for girls.

Table 3 and Fig. 3 show height SDS minus TH-SDS at the start of rhGH treatment, onset of puberty, mid-puberty, and NAH.

**Table 3.** Height SD score (SDS) minus target height–SDS was assessed at 4 critical stages during recombinant human growth hormone treatment

Height-SDS – TH-SDS		GHcont	GHstop	*P*
Start rhGH treatment	Mean (SD)	−1.95 (0.60)	−2.04 (0.53)	.37
Onset of puberty	Mean (SD)	−0.30 (0.66)	−0.24 (0.69)	.63
Mid-puberty	Mean (SD)	−0.02 (0.69)	0.13 (0.78)	.26
NAH	Mean (SD)	−0.17 (0.60)	−0.18 (0.62)	.96

Abbreviations: GH, growth hormone; GHcont, continued recombinant human growth hormone; GHstop, discontinued recombinant human growth hormone; NAH, near adult height; rhGH, recombinant human growth hormone; SDS, SD score; TH, target height.

Several patients did not meet the mid-puberty Tanner genital or breast stage criteria at inclusion. Specifically, this included 2 boys with Tanner stage G2 but with TV greater than 12 mL and BA greater than 13 years; 11 boys with Tanner stage G5 but TV between 12 and 20 mL and BA between 13 and 16 years; and 1 girl with Tanner stage M2 but a BA of 11.7 years. In boys, TV is considered a more reliable indicator of pubertal development than Tanner genital stage. After careful analysis, it was determined that these patients met the other inclusion criteria (appropriate TV and/or BA).

In boys, puberty started at age 12.0 (1.2) years in the GHcont group vs 12.04 (1.2) years in the GHstop group. Girls started puberty at age 10.9 (1.0) years in the GHcont group vs 10.7 (1.28) years in the GHstop group. The age of puberty onset was not significantly different between groups and sexes.

**Figure 4.**
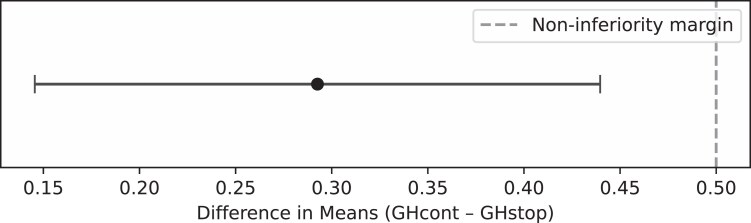
Noninferiority plot of near adult height–SD score (NAH-SDS) minus predicted adult height (PAH)-SDS, showing the 95% CI of the difference between the 2 groups (continued recombinant human growth hormone, rhGH [GHcont] – discontinued rhGH [GHstop]).

For both groups the expected height gain at inclusion was calculated based on the prediction model (36). The effectively attained height gain in both groups was compared with the predicted one. In the GHcont group the sample mean difference was −0.22 ± 0.37 SDS, t(42) = −3.85; *P*<.01. In the GHstop group the sample mean difference was −0.51 ± 0.45 SDS, t(79) = −10.76; *P*<.01. As shown in Table 4 and Fig. 4, the 95% CI of the difference excluded a difference greater than 0.5 SD that would have indicated a disadvantage for the group that discontinued rhGH in mid-puberty.

**Table 4.** Difference in near adult height–SD score (SDS) minus predicted adult height–SDS in both groups

GHcont (N = 43)	GHstop (N = 80)	*t* value	95% CI of difference
−0.22 (0.37)	−0.51 (0.45)	3.684	(0.14-0.45)

Abbreviations: GH, growth hormone; GHcont, continued recombinant human growth hormone; GHstop, discontinued recombinant human growth hormone.

Throughout the study period, 10 adverse events in 9 patients occurred, all classified as unrelated or likely unrelated. The following events were reported: psychosocial problems not otherwise specified, migraine, joint pain, upper respiratory tract infection, palpitations, COVID-19 infection, heavy menstrual bleeding, suspected spondyloarthropathy, a fifth metacarpal fracture, and a fifth metatarsal fracture. Additionally, 3 serious adverse events occurred in 3 patients; all resolved completely and were classified as unrelated or likely unrelated: testicular torsion requiring orchidopexy, severe dehydration from food poisoning during vacation abroad requiring hospital admission and intravenous rehydration, and acute appendicitis requiring surgery.

## Discussion

In this multicenter, prospective patient preference study, we evaluated whether stopping rhGH treatment at mid-puberty is noninferior as compared to continuation of rhGH treatment until NAH in adolescents diagnosed with childhood IIGHD who tested GH sufficient at mid-puberty. The mean differences in height were minimal, with a difference of 0.13 SDS (∼0.8 cm) in NAH between the groups and 0.006 SDS for NAH adjusted for TH, a difference that is neither statistically significant nor clinically relevant. Furthermore, the expected height gain at inclusion, based on the prediction models, did not show a statistically significant disadvantage for those who stopped rhGH treatment at mid-puberty, as the 95% CI of the difference excluded a difference greater than 0.5 SD (∼3 cm), the cutoff that is considered to be statistically significant and clinically relevant. Our findings indicate that discontinuing rhGH treatment at mid-puberty in previously IIGHD adolescents does not adversely affect AH outcome.

While research on rhGH withdrawal during puberty is limited, various authors agree that retesting GH secretion is essential, especially in mild to partial GHD cases, to reassess the diagnosis of IIGHD (8, 9, 21). Two studies investigated GH retesting during puberty in children with IIGHD (9, 21). Cavarzere et al (9) conducted a retrospective study comparing children with persistent GHD to those who normalized GH secretion at mid-puberty using an arginine stimulation test. Of the 80 children reassessed, 55% discontinued rhGH after testing sufficient, while 45% restarted treatment (9, 21). No significant differences in AH were observed. Similarly, Zucchini et al (21) performed a prospective study and found that 36% of children retested GH sufficient during puberty. Those who discontinued rhGH had comparable AH outcomes to those who continued treatment.

Unlike these studies, all children in our cohort retested GH sufficient, with only a subset continuing rhGH. These differences in study design highlight the importance of individualized retesting strategies and careful consideration of treatment continuation. Our study differs in several ways: Zucchini et al (21) had a smaller sample size of 69 patients in total and used a higher cutoff for the retest (10 µg/L compared to 6.7 µg/L in our study), and patients were retested at a younger age and earlier pubertal stage (boys at TV 6-12 mL and girls at breast development stages 2-3). Cavarzere et al and Zucchini et al both compared GHD patients with GH-sufficient patients (9, 21). The unique aspect of our study is that we exclusively followed GH-sufficient patients to specifically assess the effect of withdrawing rhGH treatment within this group. The previously mentioned studies advocate for retesting during mid-puberty and discontinuing treatment if the test results are normal. This contrasts with studies that recommend increasing the rhGH dose during puberty, and with the US Food and Drug Administration's approval of higher dosing based on an RCT that demonstrated increased height velocity and greater AH with a dose of 0.7 mg/kg/week (37). Since in healthy children puberty is associated with a strong increase in GH secretion, several studies were carried out to investigate the potential benefit of doubling the rhGH dose in puberty, but compared to the standard dose, the results are inconclusive (38-40). The consensus panel of the Pediatric Endocrine Society recommends against routinely increasing rhGH dose during puberty due to potential risks of GH excess, untested adverse effects, and cost concerns (20).

The age of puberty onset was similar between the groups and comparable with previous studies (41, 42). Most growth during puberty was achieved by mid-puberty, and continuation of rhGH treatment did not significantly enhance pubertal growth compared to discontinuation. Additionally, there was no significant difference in TPG between the groups. The TPG slightly exceeded that reported in the international literature and is slightly lower than our Dutch national means (girls by 2 cm and boys by 5 cm) (35, 43, 44).

The prediction model showed an overprediction both in the GHcont and GHstop group. One of the reasons is potentially the poor prediction for girls (*R^2^* of 18%) and, to a lesser extent, for boys (*R^2^* of 48%), which resulted in statistically significant differences between the groups (mean difference of 0.3 SD [−0.22 vs −0.51], ∼2.1 cm for boys and 1.9 cm for girls at age 21 years). However, in the context of noninferiority, it is crucial to consider the 95% CI of the mean difference. This spread (0.14-0.45) falls below the agreed-on threshold of 0.5 SD, which is also described in the literature as clinically relevant (45).

The decision to initiate rhGH treatment for IIGHD patients has become standard care, despite the lack of clear literature and absence of RCTs. A case-control study from India involving around 50 participants with GHD (GH peak <10 µg/L; including IIGHD and multiple pituitary hormone deficiency, excluding syndromes, chromosomal, and acquired causes) showed that untreated individuals remained at −3.5 SD in height, while those treated with rhGH reached their TH range (46). Other limited data of untreated individuals with isolated GHD show severe height deficits in untreated GHD patients (47-49). These comparative studies have shown that treated groups, including those with IGHD and idiopathic short stature, generally achieve taller AH outcomes, reaching their THs, while untreated children often have significantly shorter AHs (47-50). On the other hand, Carel et al (51) observed that long-term rhGH treatment does not provide a clear benefit for many IIGHD patients, as pubertal delay and potential for spontaneous catch-up must be considered when assessing the treatment's effectiveness and cost-effectiveness. Additionally, as stated by Yuen et al (52), the reliability and reproducibility of GH tests are critical factors, as current testing methods may be inaccurate, challenging to perform, and imprecise. Various factors further complicate the interpretation of test results, including individual patient characteristics, differences in peak GH cutoffs, testing time points, and assay heterogeneity. Importantly, the historical use of a peak GH cutoff of <10 ng/mL has been debated, as this threshold may lead to overdiagnosis and unnecessary treatment in some children. Therefore, interpretation of GHSTs should not rely solely on a single numerical cutoff but should incorporate clinical context, auxological data, and additional biochemical markers (52, 53).

Based on the results of our pilot study in 4 centers, the Dutch Growth Hormone Advisory Board developed the current prospective patient preference study combined with a retrospective historical cohort. This approach was chosen to account for potential bias in the baseline data of the 2 groups. This design, while statistically less robust, was chosen due to concerns about patient recruitment. Brettell et al (45) conducted the first RCT on GHD reversal, involving 138 children from clinics in the United Kingdom and Austria. These patients were initially diagnosed with IIGHD but showed reversal on early retesting, after which they were randomly assigned to either continue or discontinue rhGH treatment. Unfortunately, the study was discontinued due to insufficient participant recruitment (54), highlighting the challenges of investigating this topic within the framework of an RCT and thereby supporting the rationale for our study design.

We did not evaluate metabolic effects and outcomes related to body composition, muscular strength, or bone health (4, 5). While this may be seen as a limitation, it is important to note that, given the normal peak GH levels observed during retesting in our cohort, it is highly unlikely that there would be significant differences in these outcomes between groups.

With our hypothesis confirmed—withdrawing rhGH treatment from mid-puberty onward in adolescents with transient IIGHD is noninferior to continuing rhGH treatment until NAH—the guideline on rhGH treatment will be adapted accordingly. Because of the strict control of the initiation and follow-up of rhGH treatment by the Dutch Growth Research Foundation, and the limitation of prescribers to pediatric endocrinologists, it will be easy to implement the results of the study in clinical practice, ensuring compliance with this revised guideline will be close to 100% (22). This will lead to a 2- to 3-year reduction of the duration of rhGH treatment in most children with IIGHD, and a substantial decrease of medical consumption (daily rhGH injections, outpatient clinic visits, laboratory tests, and x-rays) and health-care budget.

## Data Availability

Some or all datasets generated during and/or analyzed during the current study are not publicly available but are available from the corresponding author on reasonable request.

## Clinical Trial Information

Trial registration number CCMO ID: NL57916.029.16; Toetsingonline ID: NL-OMON55366. (https://onderzoekmetmensen.nl/en/trial/55366) (registered October 26, 2016).

## Abbreviations

AH: adult height
BA: bone age
BMI: body mass index
GH: growth hormone
GHcont: continued recombinant human growth hormone
GHD: growth hormone deficiency
GHRH: growth hormone–releasing hormone
GHstop: discontinued recombinant human growth hormone
GHSTs: GH stimulation tests
IGF-I: insulin-like growth factor-I
IIGHD: idiopathic isolated growth hormone deficiency
NAH: near adult height
RCT: randomized controlled trial
rhGH: recombinant human growth hormone
TH: target height
TPG: total pubertal growth
TV: testicular volume
